# Supplementary material for: Unexpected events trigger task-independent signaling in VIP and excitatory neurons of mouse visual cortex
Source: iScience. 2025 Jan 2;28(2):111728. doi: 10.1016/j.isci.2024.111728 (PMC11787536; doi:10.1016/j.isci.2024.111728)
Supplement: Document S1. Figures S1–S6 and Tables S1–S3 [file mmc1.pdf]

iScience, Volume *28*

Supplemental information

Unexpected events trigger task-independent signaling in VIP and excitatory neurons of mouse visual cortex

Farzaneh Najafi, Simone Russo, and Jérôme Lecoq

Supplementary material

**Excitatory, V1 image responses**	one-way ANOVA: F= 16.3, p<0.001; TUKEY HSD: p<0.01 for all pairwise comparisons except layer 1-2/3 and layer 1-4.
**Excitatory, LM image responses**	TUKEY HSD: p>0.05 for all pairwise comparisons, except for layer 2/3-5: p=0.001.
**SST, V1 image responses**	one-way ANOVA: F=12.5, p<0.001; TUKEY HSD: p<0.05 for layer1-2/3, layer 1-4, and layer 4-5 pairwise comparisons.
**SST, LM image responses**	one-way ANOVA: F= 1.9, p=0.14.
**VIP, V1 image responses**	one-way ANOVA: F=3.0, p=0.03; yet TUKEY HSD: p>0.05 for all pairwise comparisons.
**VIP, LM image responses**	one-way ANOVA: F=2.4, p=0.07. VIP, V1 omission responses: one-way ANOVA: F=3.0, p=0.03; yet TUKEY HSD: p>0.05 for all pairwise comparisons. VIP, LM omission responses: one-way ANOVA: F=1.6, p=0.19.

Table S1. Statistical tests for VIP, SST, and excitatory neuron responses to image presentations and omissions. Related to Figure 1.

SLC - images

ANOVA

statistic=974.9242425360012, pvalue=2.053417877366087e-182

Posthoc

Group1	Group2	p
0	1	<0.001
0	2	0.0045
0	3	<0.001
1	2	<0.001
1	3	0.17
2	3	<0.001

Ranksum

Group	p	effect size
1	0.5	-0.000559821
2	<0.001	0.005208667
3	0.8	-0.000677232
4	<0.001	-0.006407717

SLC - omissions

ANOVA

statistic=924.6397775278477, pvalue=2.0365188317079515e-178

Posthoc

Group1	Group2	p
0	1	<0.001
0	2	0.9
0	3	<0.001
1	2	<0.001
1	3	0.9
2	3	<0.001

Ranksum

Group	p	effect size
1	<0.001	-0.007043246
2	<0.001	-0.010607699
3	<0.001	-0.005858144
4	<0.001	-0.011777935

SST - images

ANOVA

statistic=562.5678946813928, pvalue=2.34542498531712e-142

Posthoc

Group1		
0	1	0.26
0	2	<0.001
0	3	<0.001
1	2	<0.001
1	3	<0.001
2	3	<0.001

Ranksum

Group	p	effect size
1	<0.001	-0.020148597
2	<0.001	0.008925503
3	<0.001	-0.018708875
4	<0.001	-0.015717922

SST - omissions

ANOVA

statistic=388.69757476891175, pvalue=1.3433542133282506e-117

Posthoc

Group1		
0	1	<0.001
0	2	<0.001
0	3	<0.001
1	2	<0.001
1	3	<0.001
2	3	<0.001

Ranksum

Group		
1	<0.001	-0.021273879
2	<0.001	-0.032506687
3	<0.001	-0.010707941
4	<0.001	-0.061329306

LEGEND	
group 1	LM superficial
group 2	LM deep
group 3	V1 superficial
group 4	V1 deep

VIP - omissions

ANOVA

statistic=152.09786442559385, pvalue=1.4251507357988139e-65

Posthoc

Group1		
0	1	<0.001
0	2	<0.001
0	3	<0.001
1	2	<0.001
1	3	0.0058
2	3	<0.001

Ranksum

Group		
1	<0.001	-0.054864618
2	<0.001	-0.017996513
3	<0.001	-0.068608776
4	0.009	-0.005409973

Table S2. Statistical tables for TCA analysis. Related to Figure 3.

Number of components	Celltype	Data	Error	Similarity
5	Excitatory	Original	0.9401	0.8879
		Shuffled	0.9681	0.8673
	SST	Original	0.7695	0.9227
		Shuffled	0.8365	0.9067
	VIP	Original	0.774	0.918
		Shuffled	0.8838	0.8886
10	Excitatory	Original	0.9084	0.8265
		Shuffled	0.9366	0.8038
	SST	Original	0.67	0.8695
		Shuffled	0.7481	0.858
	VIP	Original	0.6991	0.8515
		Shuffled	0.8238	0.8195

Table S3. TCA error and similarity values for different number of TCA components, cell types, and for original vs shuffled data. Related to Figure 3.

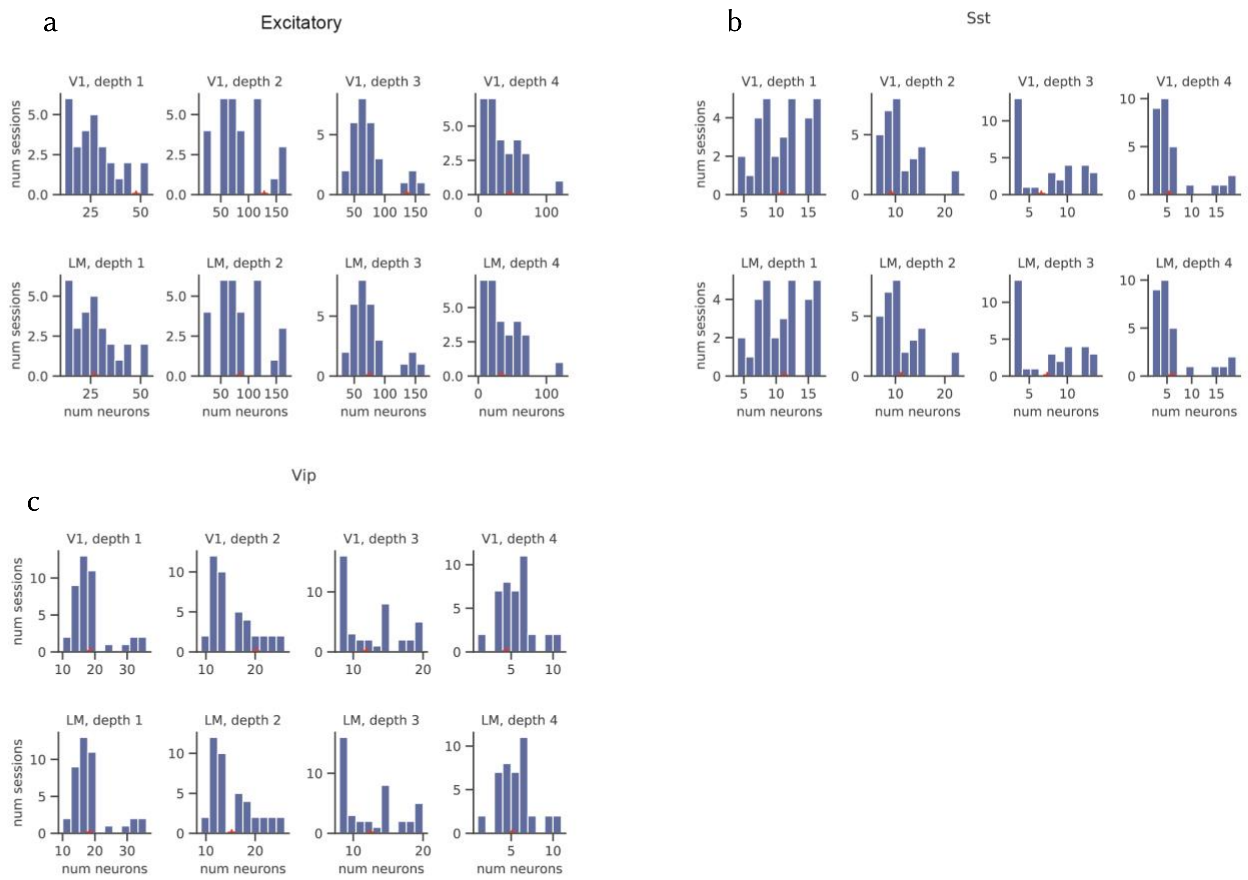

Figure S1. Number of neurons per depth and area, for each cell type. Distribution of number of neurons recorded simultaneously across depths of V1 (top) and LM (bottom), in all recording sessions, for excitatory **(a)**, SST **(b)**, and VIP **(c)** mice. Note that our goal in this study was to chronically record the activity of distinct excitatory and inhibitory cell types, from the same field of view and across multiple sessions. Like in other studies involving the original Mesoscope[1,2], our system can record from a much larger number of neurons, however, our biological experiments were not designed for that purpose. Related to Figure 1.

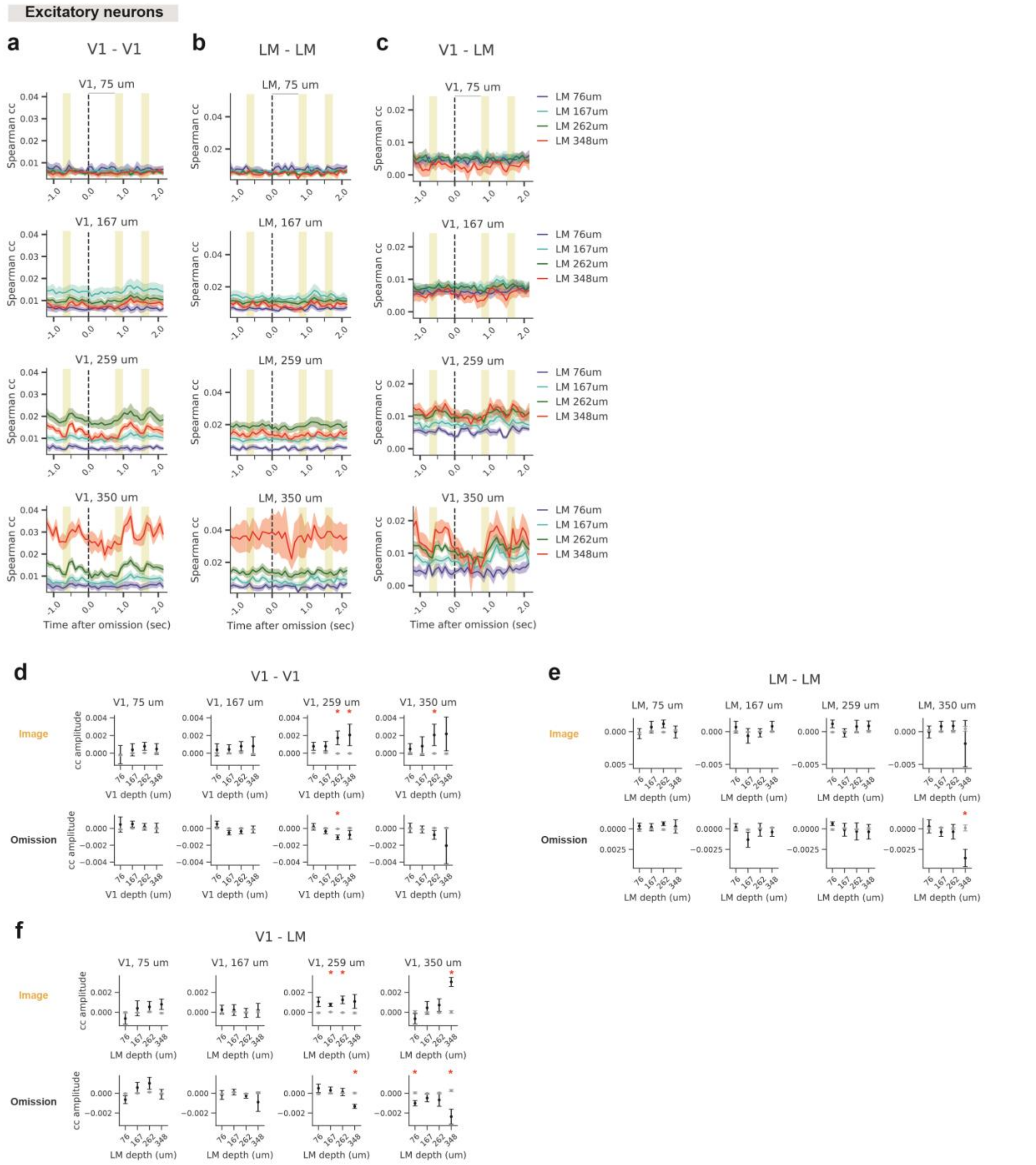

Figure S2. Correlation of excitatory neurons within V1, within LM, and across V1-LM, during images and omissions. **a-c** Spearman correlation coefficients computed, at different moments in the trial, between activities of excitatory neurons located in different depths of V1-V1 (a), LM-LM (b), and V1-LM (c). **d-f** Change in correlation coefficients during images (top) and omissions (bottom) relative to the baseline correlation coefficient, computed for the real data (black), and trial shuffled data (gray). Correlation coefficients were quantified over 500 ms after images, and 750 ms after omissions. Red stars indicate statistical significance (two-sided t-test, real vs. shuffle data; p<0.05). Traces and error-bars: mean +/- SEM; n = 8 mice. Related to Figure 2.

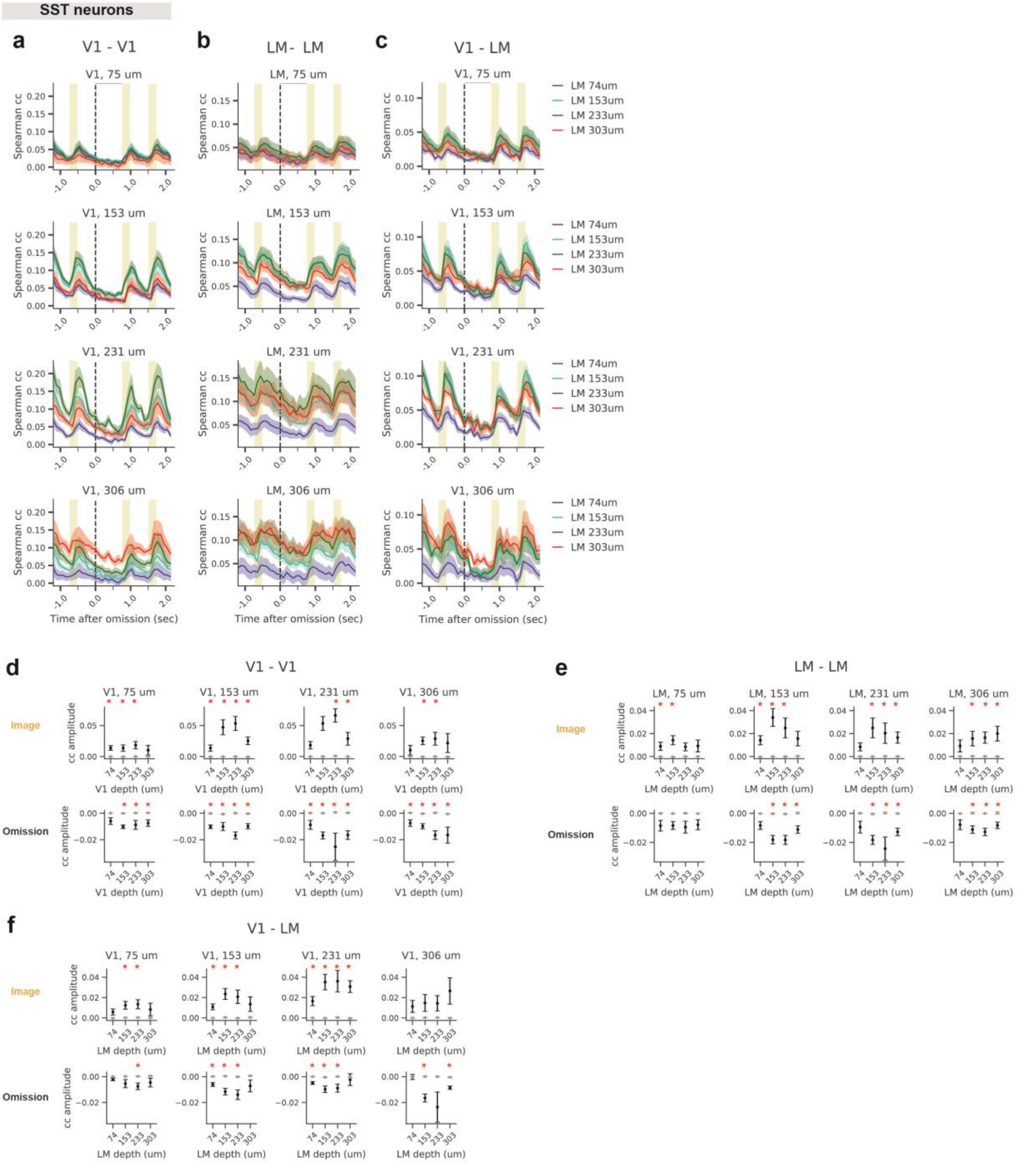

Figure S3. Correlation of SST neurons within V1, within LM, and across V1-LM, during images and omissions. a-c Spearman correlation coefficients computed, at different moments in the trial, between activities of excitatory neurons located in different depths of V1-V1 (a), LM-LM (b), and V1-LM (c). **d-f** Change in correlation coefficients during images (top) and omissions (bottom) relative to the baseline correlation coefficient, computed for the real data (black), and trial shuffled data (gray). Correlation coefficients were quantified over 500 ms after images, and 750 ms after omissions. Red stars indicate statistical significance (two-sided t-test, real vs. shuffle data; p<0.05). Traces and error-bars: mean +/- SEM; n = 6 mice. Related to Figure 2.

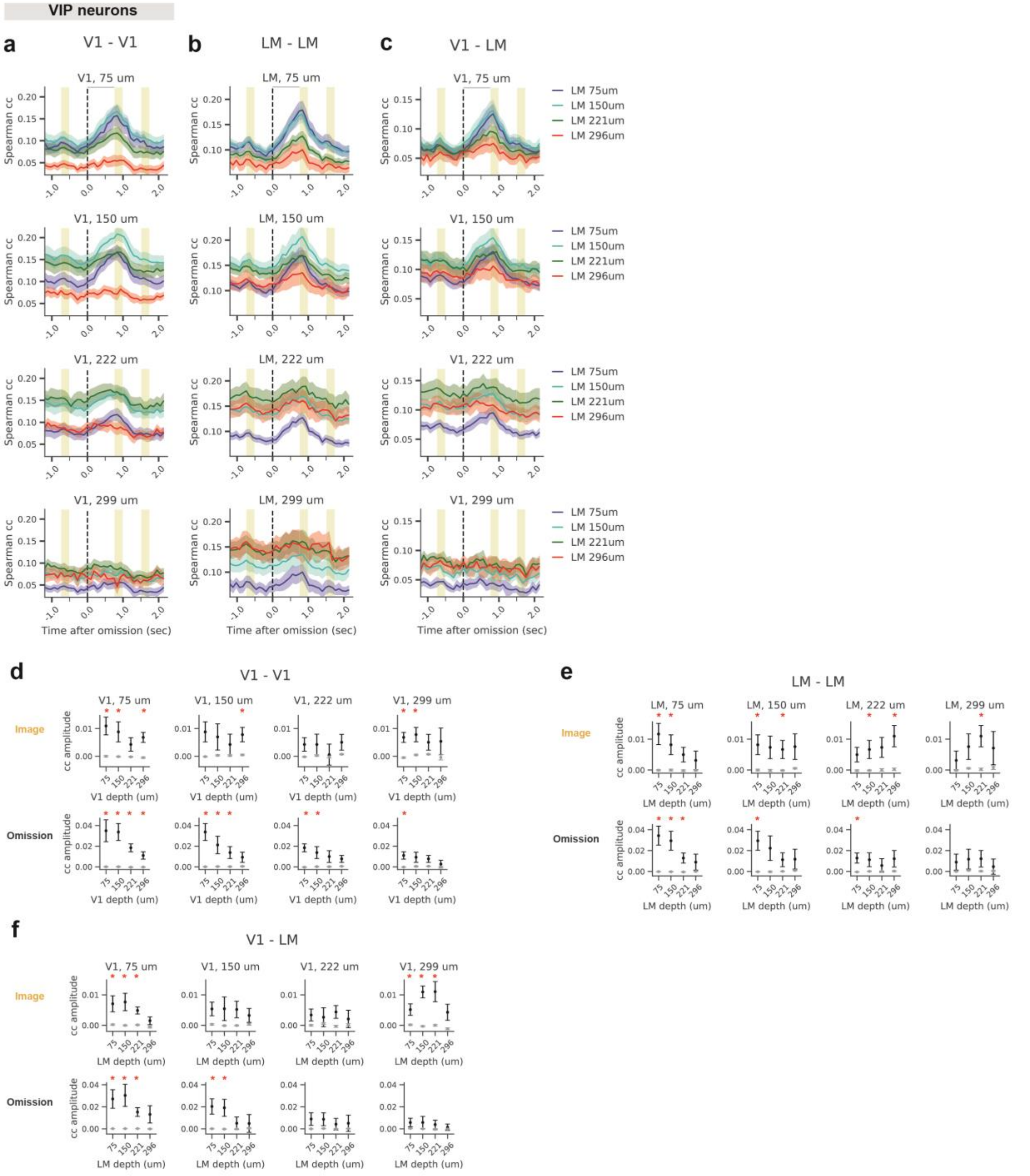

Figure S4. Correlation of VIP neurons within V1, within LM, and across V1-LM, during images and omissions. a-c Spearman correlation coefficients computed, at different moments in the trial, between activities of excitatory neurons located in different depths of V1-V1 (a), LM-LM (b), and V1-LM (c). **d-f** Change in correlation coefficients during images (top) and omissions (bottom) relative to the baseline correlation coefficient, computed for the real data (black), and trial shuffled data (gray). Correlation coefficients were quantified over 500 ms after images, and 750 ms after omissions. Red stars indicate statistical significance (two-sided t-test, real vs. shuffle data; $p<0.05$). Traces and error-bars: mean +/- SEM; n = 9 mice. Related to Figure 2.

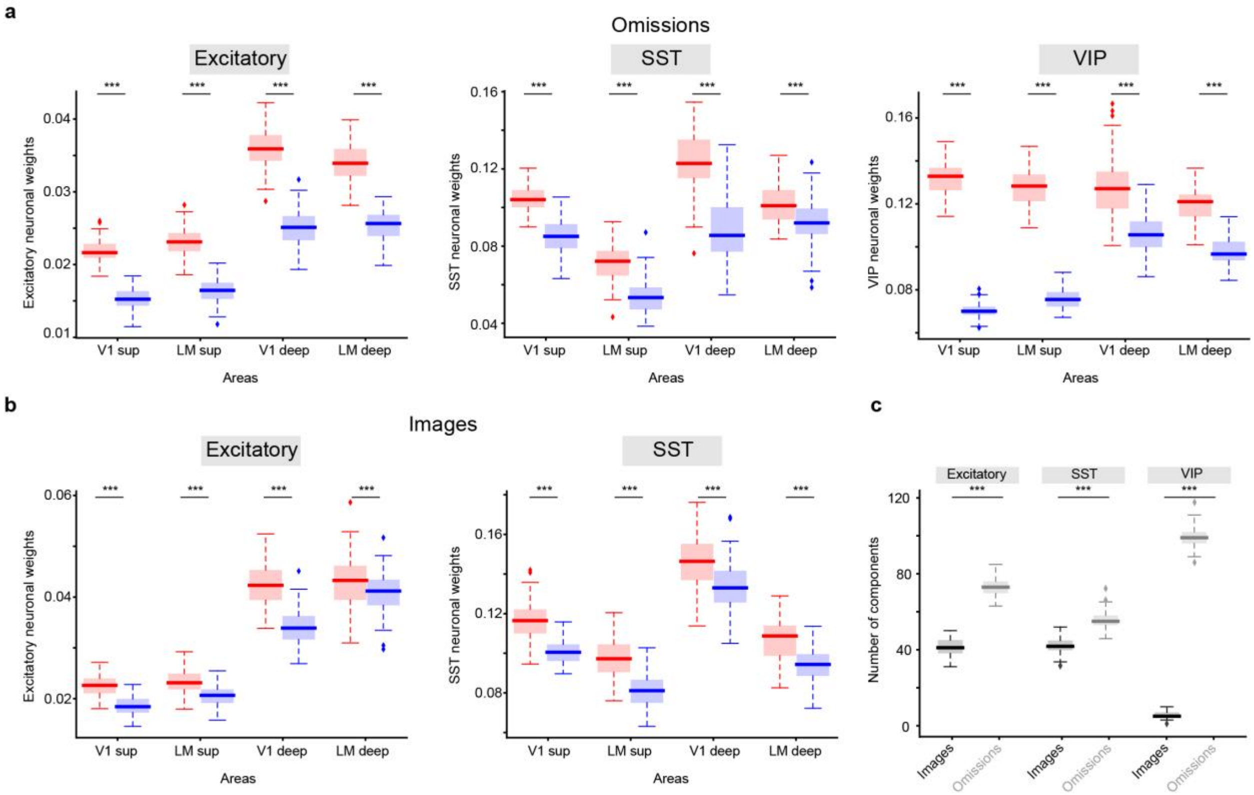

Figure S5. TCA with 10 components. a, b, and c depict the results for the TCA analysis for 10 components corresponding to Figure 3D,E,F, respectively. Related to Figure 3.

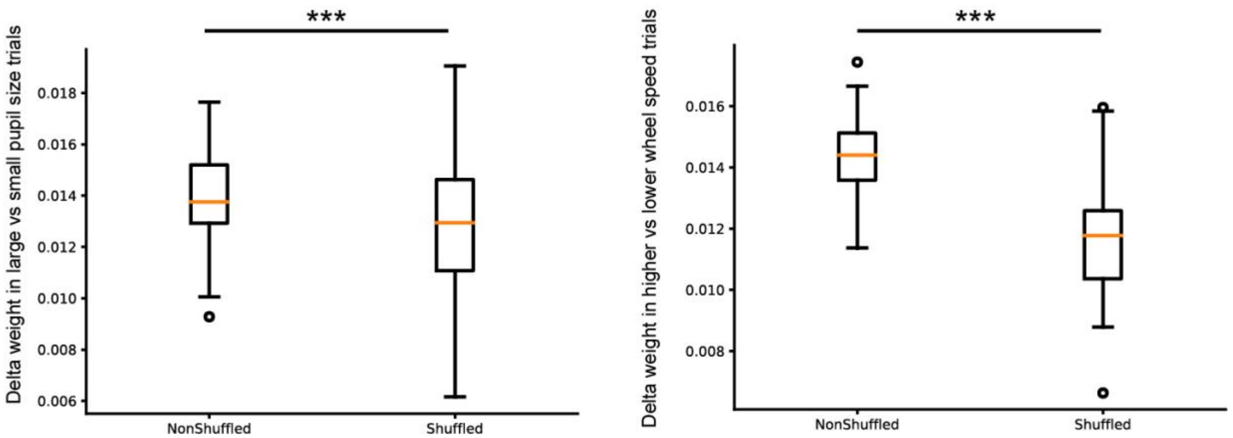

Figure S6. The non-task signals that emerge in VIP neurons during omissions encode behavioral features. The left panel depicts the difference between the weight of trials with large and small pupil size in the original and shuffled VIP data (Wilcoxon ranksum test; p<0.001). The right panel depicts the same variable for trials with higher and lower wheel speed (Wilcoxon ranksum test; p<0.001). Related to Figure 3.
